# *Ex Vivo* Expanded Human Regulatory T Cells Delay Islet Allograft Rejection via Inhibiting Islet-Derived Monocyte Chemoattractant Protein-1 Production in CD34^+^ Stem Cells-Reconstituted NOD-*scid IL2rγ^null^* Mice

**DOI:** 10.1371/journal.pone.0090387

**Published:** 2014-03-03

**Authors:** Fang Xiao, Liang Ma, Min Zhao, Guocai Huang, Vincenzo Mirenda, Anthony Dorling, Robert Lechler, Giovanna Lombardi

**Affiliations:** 1 Medical Research Council (MRC) for Transplantation, King's College London, London, United Kingdom; 2 Department of Diabetes & Endocrinology, King's College London, London, United Kingdom; Children's Hospital Boston/Harvard Medical School, United States of America

## Abstract

Type 1 diabetes mellitus (T1DM) is an autoimmune disease caused by immune-mediated destruction of insulin-secreting β cells of the pancreas. Near complete dependence on exogenous insulin makes T1DM very difficult to control, with the result that patients are exposed to high blood glucose and risk of diabetic complications and/or intermittent low blood glucose that can cause unconsciousness, fits and even death. Allograft transplantation of pancreatic islets restores normoglycemia with a low risk of surgical complications. However, although successful immediately after transplantation, islets are progressively lost, with most of the patients requiring exogenous insulin within 2 years post-transplant. Therefore, there is an urgent requirement for the development of new strategies to prevent islet rejection. In this study, we explored the importance of human regulatory T cells in the control of islets allograft rejection. We developed a pre-clinical model of human islet transplantation by reconstituting NOD-*scid IL2rγ^null^* mice with cord blood-derived human CD34^+^ stem cells and demonstrated that although the engrafted human immune system mediated the rejection of human islets, their survival was significantly prolonged following adoptive transfer of *ex vivo* expanded human T_regs_. Mechanistically, T_regs_ inhibited the infiltration of innate immune cells and CD4^+^ T cells into the graft by down-regulating the islet graft-derived monocyte chemoattractant protein-1. Our findings might contribute to the development of clinical strategies for T_reg_ therapy to control human islet rejection. We also show for the first time that CD34^+^ cells-reconstituted NOD-*scid IL2rγ^null^* mouse model could be beneficial for investigating human innate immunity *in vivo*.

## Introduction

Type 1 diabetes mellitus (T1DM) is a chronic autoimmune disease in which insulin-secreting pancreatic β cells are destroyed by autoreactive T cells. It is characterized by dysregulation of blood glucose caused by β -cell insufficiency. Among the available treatments, human islet transplantation has emerged as a promising approach that restores glycemic stability even if full discontinuation of insulin treatment is not practical [1]–[3]. Transplanted islets, which consist of small clusters of cells containing insulin-producing β-cells, however provoke the same immunological responses as solid organ transplants, and necessitate immunosuppressive therapy to reduce immune-mediated rejection [4]. Immunosuppressive drugs have severe side effects, and they are toxic to β-cells as well as being ineffective at preventing late-stage allograft rejection [5], [6]. To achieve durable success in islet transplantation, there is a need to develop applicable strategies for immunomodulation in order to suppress T cell-mediated alloimmune responses [7]–[10].

CD4^+^CD25^hi^FoxP3^+^ regulatory T cells (T_regs_) have been shown to be important for the control of autoimmunity and maintenance of allograft tolerance [7]. In humans, normally only 5–10% of peripheral blood CD4 T cells are natural-occurring regulatory T cells [11] and thus large-scale *ex vivo* expansion is required for therapeutic application [12]. We and others have recently shown that large-scale *ex vivo* expanded human T_regs_ maintain their immunosuppressive capacity and are stable *in vitro* and *in vivo* under inflammatory conditions and are safe for clinical use [13]–[16]. This raises the possibility that T_regs_ may be used in islet transplantation to protect human grafted islets from alloimmune rejection without the side effects of systemic immunosuppression.

The recent development of ‘humanized’ mice has allowed the *in vivo* investigation of human immune responses [17]–[20]. In transplantation models, we and others have demonstrated that *ex vivo* expanded human T_regs_ can prevent the development of transplant arteriosclerosis [21] and skin allograft rejections [22] in peripheral blood mononuclear cells (PBMC)-reconstituted humanized mouse models [23]. The protective function of adoptively transferred human T_regs_ has also been investigated in PBMC-reconstituted humanized mouse transplanted with porcine islets. More recently, Douglas *et al*. have shown that human T_regs_ can prevent human islet transplant rejection by inhibiting T cell differentiation in PBMC-reconstituted Balb/cRag2^−/−^γc^−/−^ mouse model [24]. In the present study, we adoptively transferred *ex vivo* expanded human T_regs_ into CD34^+^ stem cells-reconstituted NOD-*scid IL2rγ^null^* (NSG) mice to determine whether T_regs_ can protect grafted human islets from immune-mediated rejection, focusing primarily on the effects of T_regs_ on the innate immune responses.

## Materials and Methods

### Ethics statements

Use of human peripheral blood from healthy volunteers (REC Ref: 09/H0707/86), cord blood from full-term deliveries (REC Ref: 08/H0802/160) and islets from cadaveric donors (REC Ref: 05/MER09/48) has been approved by the National Research Ethics Service Committee London - Westminster, UK. Blood donors and the relatives of islet donors gave written informed consent for scientific use of the human materials. All consent procedures were approved by the National Research Ethics Service Committee London - Westminster, UK.

All animal experiments were conducted in accordance with UK Research Councils' and Medical Research Charities' guidelines on Responsibility in the Use of Animals in Bioscience Research, and the Home Office Animals Scientific Procedures Act (1986) under a UK Home Office license (PPL 70/7302). Anesthetics and analgesics were administered to minimize or eliminate the pain and distress appropriately.

### Isolation of CD34^+^ stem cells from human umbilical cord blood

Cord blood was supplied by the NHS Cord Blood Bank (Colindale, London, UK). Mononuclear cells were isolated using density gradient centrifugation over Ficoll-Paque (GE Healthcare, Hatfield, UK) and enriched for CD34^+^ cells by positive immunomagnetic isolation according to the manufacturer's instructions (Miltenyi Biotech, Surrey, UK).

### Reconstitution of mice with human stem cells

**Figure 1:**
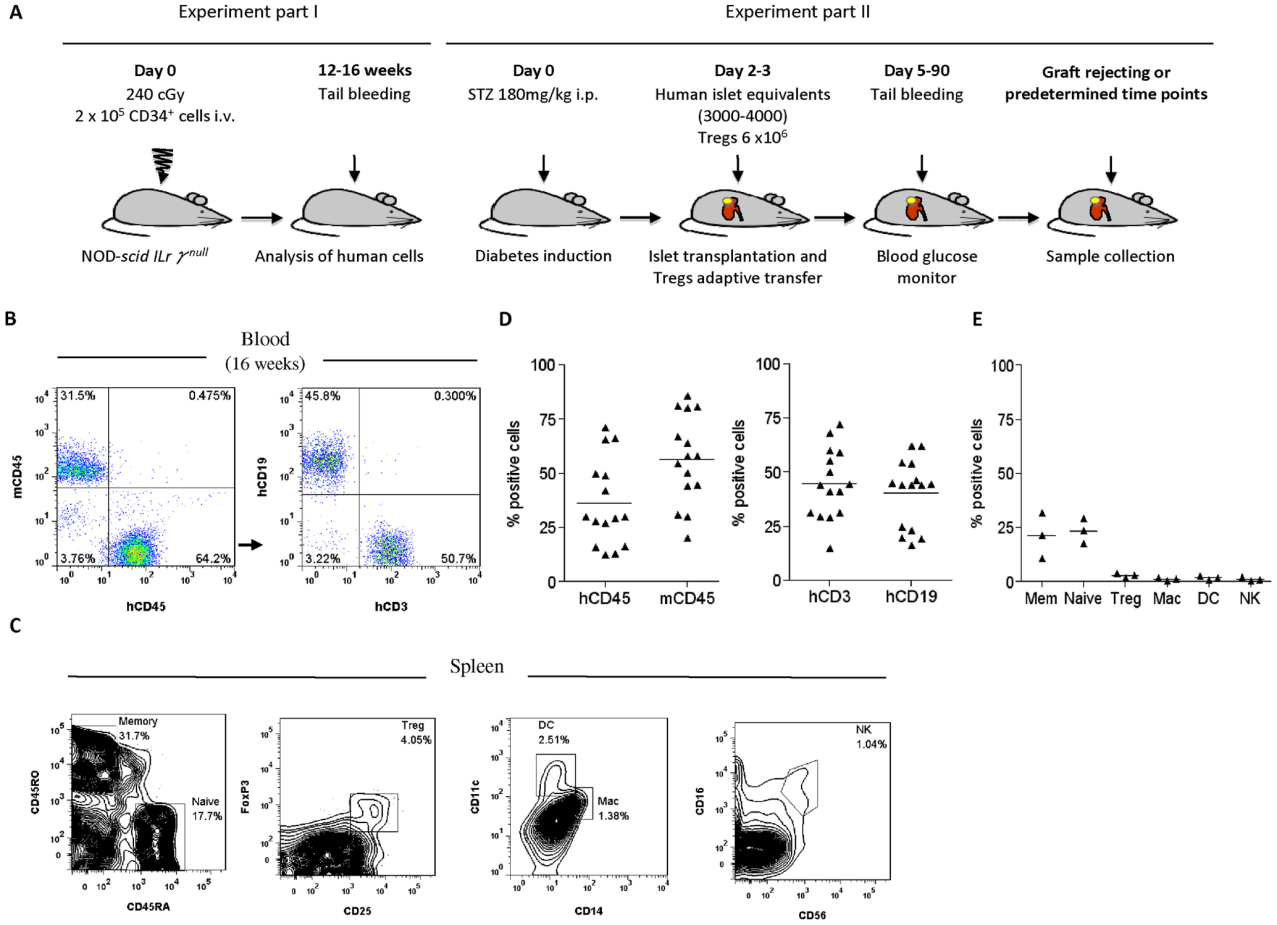
Reconstitution of NSG mice with human CD34^+^ stem cells. (A) Strategy for generation of humanized mouse model of islet transplantation. (B) A representative FACS profile of human CD45^+^, human CD19^+^ and human CD3^+^ cells in peripheral blood of hu-NSG mice, 12–16 weeks after transfer of CD34^+^ cells (n = 15). Human CD19^+^ B cells and CD3^+^ T cells were identified in the gate for human CD45^+^ cells. (C) Splenocytes from hu-NSG mice were analyzed by FACS for human CD45RA^+^ (naïve T cells), human CD45RO^+^ (memory T cells), human CD4^+^CD25^+^FoxP3^+^ (Tregs), human CD11c^+^ (dendritic cells), human CD14^+^ (macrophages) and human CD16^+^CD56^+^ (NK cells) expression. (D–E) Data are summarized. Plotted lines represent the mean (n = 15, D; n = 3, E).

NSG mice (The Jackson Laboratory) were bred and maintained in the Biological Services Unit of King's College London under specific pathogen-free conditions. Mice of 4–6 week old were irradiated with 240 cGy of γ-rays and received intravenous injection of 2×10^5^ CD34^+^ stem cells within 24 hours (**Fig. 1A**). For simplicity, the CD34^+^ cells-reconstituted NSG mice are hereafter referred to as hu-NSG mice and NSG mice lacking CD34^+^ cells injection are referred to as NSG mice.

### Preparation of human regulatory T cells

Human leukocyte cones were obtained from the National Blood Transfusion Service (Tooting, London, UK). PBMC were isolated by Lymphocyte (PAA, Austria) density gradient centrifugation. CD4^+^CD25^+^ T cells (T_regs_) were isolated from PBMC using a CD4^+^CD25^+^ Regulatory T Cell Isolation Kit (Miltenyi Biotec) according to the manufacturer's instructions. The CD4^+^CD25^−^ cells (T_effs_) were used as autologous responder cells for *in vitro* suppression assays. Isolated T_regs_ were expanded as previously published [14]. Briefly, T_regs_ were cultured in X-vivo 15 medium (Lonza, Slough, UK) supplemented with 5% of human AB male pooled serum (HS, Biosera, London, UK), in the presence of recombinant IL-2 (1000 IU/mL, Proleukin-Novartis, Surrey, UK), rapamycin (100 nM) (Sigma, Surrey, UK) and Human T-Expander CD3/CD28 Dynabeads (CD3/CD28 beads, Invitrogen, Paisley, UK) in a 1∶1 cell to bead ratio for three rounds of expansion, 8–10 days each. After the third round of expansion, the beads were removed and the T_regs_ were rested for 5–7 days in medium containing low concentration of IL-2 (20 IU/mL) before being intravenously injected into the mice.

### T cell proliferation assay

Cell suspensions (1×10^5^) prepared from the spleen of hu-NSG mice were cultured in 10% FBS RPMI-1640 medium in the presence of CD3/CD28 beads at a 1∶1 bead to cell ratio. T-cell proliferation was measured after a 72 hour culture by adding ^3^H thymidine (1 µM) for the last 16 hours. In a mixed lymphocyte reaction (MLR), human CD4^+^ cells isolated from the spleen of hu-NSG mice immunized by HLA-mismatched PBMC were co-cultured with serial dilutions of irradiated [3000 gray (Gy) for 10 min] PBMC from the same donor (specific PBMC) and the PBMC from an unrelated donor (third-party PBMC). The proliferation assays were performed in a five day culture.

The suppressive activity of expanded T_regs_ was tested by co-culturing responder T cells (CD4^+^CD25^−^, T_effs_) labelled with 5 µM carboxyfluorescein succinimidyl ester (CFSE, Invitrogen) with serial dilutions of *ex vivo* expanded autologous T_regs_ in the presence of CD3/CD28 beads in a 1∶1 T_effs_ to bead ratio in RPMI-1640 medium containing 10% of human AB serum for 5 days. Proliferation was analyzed by flow cytometry. The percentage of proliferative T_effs_ in the absence of T_regs_ was taken as 100% proliferation.

### Islet transplantation and adoptive transfer of T_regs_

Hu-NSG and NSG mice were rendered diabetic (blood glucose level≥20 mM) by a single intraperitoneal (i.p.) injection of streptozotocin (STZ), 180 mg/kg body weight. Human islets were obtained from King's Cell Isolation Unit (London, UK). All islets were maintained in CMRL 1066 culture medium (Invitrogen) containing 2.5% human albumin at 37°C in an atmosphere of 95% air 5% CO_2_ until transplanted. Human islet equivalents (IEQs, 3000–4000) were then transplanted under the left kidney capsule of diabetic mice that were anesthetized with pentobarbital sodium at 50 mg/kg [25]. After surgery, the mice were given subcutaneously 0.03 mg/ml of Vetergesic Multidose (buprenorphine) (Alstoe Ltd. Animal Health, UK) in 100 µl of sterile saline for post-operative analgesia. A total of 6×10^6^ *ex vivo* expanded human T_regs_ expressing the same HLA-DR as the engrafted CD34^+^ stem cells (**Table S1**) were injected intravenously into recipient mice immediately after islet transplantation. Blood glucose levels in recipient mice were evaluated using a blood glucose sensor (Abbott Diabetes Care Ltd., Witney, Oxon, UK). Loss of graft function was determined by blood glucose values≥20 mM.

### Nephrectomy

Selected mice with successful islet transplants were subjected to unilateral left nephrectomy to evaluate a return to hyperglycemic states. Anaesthetized mice were placed in a lateral decubitas position and a 2 cm incision was made over the left flank. The kidneys were extracorporealized. The renal artery was ligated together with the vein and ureter, prior to kidney resection. All animals were monitored closely postoperatively, and blood glucose was measured every 24 hours for 5 days.

### Flow cytometric analysis

Flow cytometric analysis of human antigens in human T_regs_ and leukocytes was performed as previously described [26]. Fluorochrome-coupled antibodies specific for human CD45, CD3, CD19, CD45RA, CD45RO, CD4, CD25, FoxP3, CD14, CD11C, CD16 and CD56 were used for analysis of the human cell engraftment in hu-NSG mice. An APC-conjugated antibody against human monocyte chemoattractant protein-1 (MCP-1) was used for the detection of MCP-1 expression in islet cells. Single cell suspensions from the draining lymph nodes (left renal) and spleens were prepared from the islet-transplanted hu-NSG mice at the time of rejection (islets alone group) or at 21 days post-transplantation (islets+T_regs_ group), and stained with antibodies against human CD4, CD25 and FoxP3. Flow cytometric data were acquired using a FACS Calibur (BD Biosciences, San Jose, CA, USA) and analyzed using FlowJo 7.5 software (TreeStar Inc.).

### Co-culture of human islets and T_regs_

The human islets were dissociated into single cells with 0.05% trypsin-EDTA. The single cells (0.5 million cells) were cultured with or without 1 million *ex vivo* expanded T_regs_ in the presence of CD3/CD28 beads in 10% HS RPMI-1640 medium for 48 hours. LPS (1 µg/mL) was then added to the cultures for an additional 24 hours. As a control, the islets were co-cultured with autologous T_effs_ under the same conditions. After the incubation period, T_regs_ or T_effs_ were positively isolated using CD4 Dynabeads (Invitrogen). The eluted fraction was used for flow cytometric analysis and real-time RT-PCR for expression of MCP-1.

### Real-time RT-PCR

PCR primers specific for human MCP-1 were designed as previously published [27] and RNA was extracted from islet grafts harvested at the time of rejection (islets alone group) or at 21 days post-transplantation (islets+T_regs_ group) and from cultured single islet cells using RNeasy Mini Kit (Qiagen). Real-time RT-PCR was performed using Brilliant SYBR Green QRT-PCR Master Mix Kit, following the manufacturer's protocol (Agilent Technologies, UK).

### Enzyme-linked immunosorbent assay (ELISA) for human IgG, IgM, insulin and complement 3 (C3)

Human IgM and IgG were measured in the serum of hu-NSG mice over 12–16 weeks after the CD34^+^ stem cells injection, and then anti-keyhole limpet hemocyanin (KLH) IgM and IgG were measured two weeks after hu-NSG mice were immunized with KLH whole protein (Sigma), using standard ELISA protocols (11).

Mouse sera taken from islet-transplanted hu-NSG mice at the time points when blood glucose was 7, 20 and 28 mM were assessed for human insulin and sera taken at the time of islet allograft rejection (blood glucose>20 mM) were determined for human C3 by ELISA kits (Millipore, Watford, UK).

### Cytokine detection

Human Th1/Th2 cytokines were determined in the supernatants of CD4^+^ T cells isolated from the spleen cells of hu-NSG mice after a 3 day stimulation by CD3/CD28 beads, and in the serum samples harvested at the time of rejection (islets alone group) or at 21 days post-transplantation (islets+T_regs_ group). A human Th1/Th2 11plex kit (eBiosciences) was used according to the manufacturer's protocol. Data acquisition was performed on a FACS Calibur (BD Biosciences) and then was analyzed using the BD Cytometric Bead Array software (BD Biosciences). The cytokines detected were IFN-γ, IL-1β, IL-2, IL-4, IL-5, IL-6, IL-8, IL-10, IL-12p70, TNF-α and TNF-β.

### Histological and immunohistochemical analysis

Graft-bearing kidneys were harvested, fixed in 10% buffered formalin, and embedded in paraffin at the time of rejection (islets alone group) or at 21 days post-transplantation (islets+T_regs_ group). Sections (5 µm) were conventionally processed and stained with hematoxylin and eosin (H&E). For characterization of cell type-specific expression of antigens, single immunofluorescence staining was used: after antigen retrieval by microwaving for 5 min in 0.01 M citrate buffer (pH 6.0), sections were blocked with 10% goat normal serum for 30 min and then incubated overnight at 4°C with primary antibodies against the following: human CD45 (1∶50; Dako, clone 2B11+PD7/26), insulin conjugated with HRP (1∶50; Abcam, clone D3E7), complement 3d (C3d) conjugated with HRP (1∶60; Dako, polyclonal), human CD4 (1∶40; Dako, clone 4B12), human CD8 (1∶60; Abcam, clone 14), human CD11b (1∶50; eBiosciences, clone ICRF44) and human CD66b (1∶50; BioLegend, clone G10F5). Diaminobenzidene and FITC- or TRITC-conjugated secondary antibodies were applied for 2 hours at room temperature. Sections were mounted in ProLong Gold Antifade Reagent with DAPI (Invitrogen). For identification of T_regs_, slides were double stained with primary antibodies: mouse anti-human CD4 and rabbit anti-human FoxP3 (1∶60; both Abcam, polyclonal). FITC-conjugated goat anti-mouse IgG and TRITC-conjugated goat anti-rabbit IgG (both Sigma) were used as secondary antibodies. Negative controls with nonspecific IgG were processed in parallel. Images were acquired using a Cooled Mono14 Bit camera (Q IMAGING, Canada) and Micro-Manager 1.3 software (University of California, USA).

### Statistics

All statistical analysis were performed using the two-tailed Student's *t*-test (unpaired) for the group data and log-rank test for the survival data (Prism 4.0; GraphPad Software, USA). Data were presented as means ± SD and *P* values<0.05 were considered significant.

## Results

### NSG mice reconstituted with human CD34^+^ stem cells are engrafted with a functional human immune system

A number of humanized mouse models have been generated for the *in vivo* study of human immune responses [28], [29]. Here, NSG mice of 4-6 weeks of age were injected with cord blood-derived CD34^+^ stem cells (**Fig. 1**A). After 3 months, a significant percentage of human cells was identified by flow cytometric analysis in the blood of NSG recipients (36%±5%) with 1∶1 ratio of T and B cells (**Fig. 1B and D**). Various subsets of immune cells were present in the spleens of these animals, including naïve T cells (CD45RA^+^), memory T cells (CD45RO^+^), T_regs_ (CD4^+^CD25^+^FoxP3^+^), macrophages (CD14^+^), dendritic cells (CD11c^+^) and NK cells (CD16^+^CD56^+^) (**Fig. 1C and E**).

To validate the function of engrafted T cells, splenocytes from hu-NSG mice were stimulated *in vitro* with anti-CD3/CD28 beads. T cells responded to polyclonal stimulation (**Figure S1** (A)) and the cytokines IL-2, TNF-α, TNF-β, IL-8 and IL-1β were detected in culture supernatants (**Table 1**). Next, human CD4^+^ T cells from the spleens of hu-NSG mice that had been immunized *in vivo* with allogeneic PBMC (specific PBMC) (**Figure S1** (B)) were re-stimulated *in vitro* with specific PBMC or with third party PBMC. These CD4^+^ T cells showed significant responses to the specific PBMC, but not to third party PBMC (**Figure S1** (B)).

**Table 1:** Cytokine production by human CD4 T cells from hu-NSG mice *in vitro*.

	Spl:CD4		AdPBMC:CD4	
Pg/mL	Control	CD3/CD28	Control	CD3/CD28
IFN-γ	ud*	ud	ud	168.5±97.27
IL-2	ud	298.58±20.26	ud	1750.18±223.93
TNF-α	ud	62.30±6.86	ud	1197.47±192.22
TNF-β	7.75±2.62**	118.62±21.35	5.98±2.45	510.9±89.35
IL-10	ud	ud	ud	201.91±39.45
IL-8	101.83±34.39	150.41±9.21	73.54±22.63	1871.14±318.04
IL-1β	2.52±1.45	0.63±0.36	3.17±1.83	10.98±6.33

Human CD4^+^ T cells were stimulated with CD3/CD28 beads for three days. Supernatants were analyzed by cytometric bead array for cytokine production (n = 3). Cytokines IL-4, IL-5, IL-6 and IL-12p70 were not detectable in the supernatants. Spl:CD4 denotes CD4 cells from the spleen of a hu-NSG mouse. AdPBMC:CD4 indicates CD4 cells from PBMC of a healthy donor. *: undetectable. **: mean ± SD.

The function of B cells in hu-NSG mice was also evaluated and both IgM and IgG were detected in sera (IgM: 76.67±1.45 µg/mL; IgG: 44.39±9.4 µg/mL) (**Figure S2** (A)) and in supernatants after culturing B cells *in vitro* in the presence of IL-2 and IL-21 (IgM: 0.37±0.013 µg/mL; IgG: 0.99±0.21 µg/mL) (**Figure S2** (A)). Furthermore, KLH-specific IgM responses, but not IgG, were induced *in vivo* after KLH immunization (**Figure S2** (B)), as previously observed [26], [30].

### Human islet allografts stimulate immune responses in hu-NSG mice

**Figure 2:**
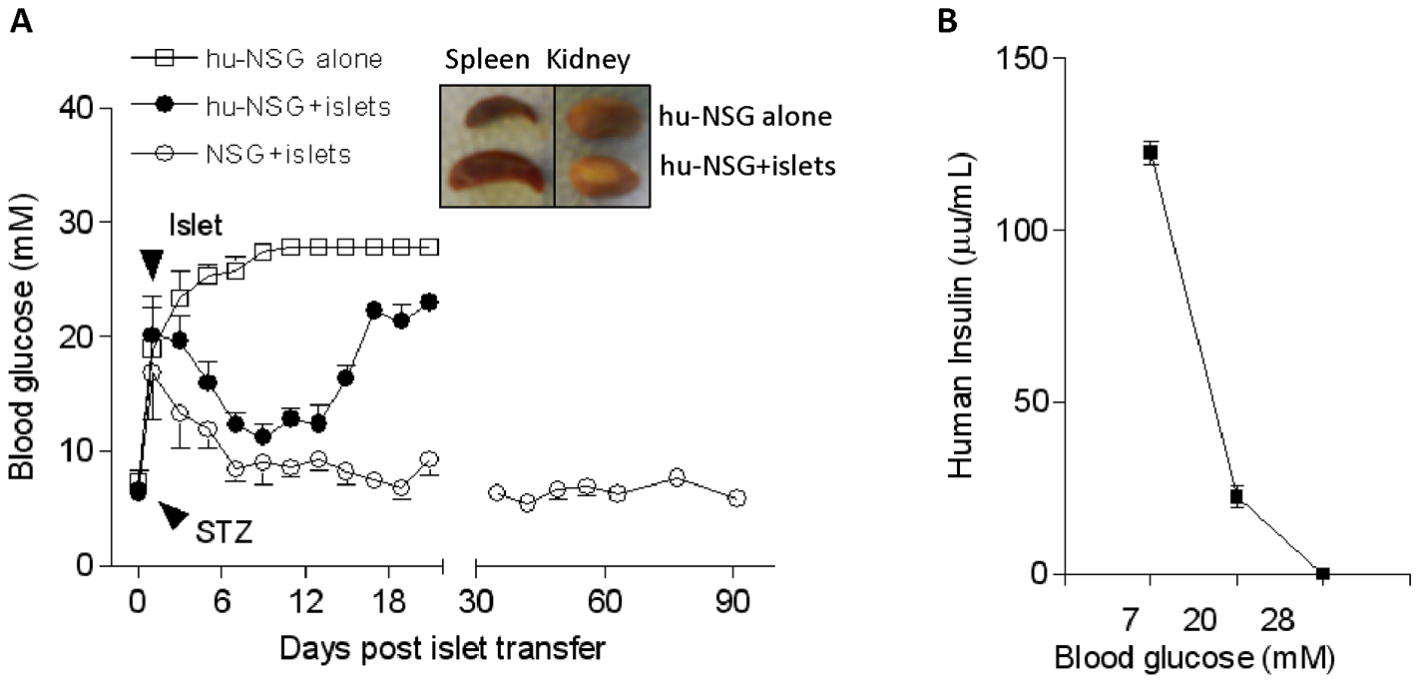
Diabetic hu-NSG mice reject the grafted human islets. (A) The function of grafted human islets was evaluated by measuring blood glucose levels. As a control, NSG mice without CD34^+^ cell reconstitution remained normoglycemic after islet transplantation. The insert shows a representative image of a kidney and a spleen from hu-NSG mice that received (bottom) or did not receive (top) islet transplants (n = 6). STZ: streptozotocin. (B) Serum samples from hu-NSG mice that were grafted with human islets were measured by ELISA for human insulin when blood glucose was 7, 20 and 28 mM (n = 3).

Having confirmed that hu-NSG mice had a functional adaptive immune response, although partially impaired [26], [30], [31], mice with successful human cell engraftment (human CD45^+^ cells>15%) were rendered diabetic. They were then transplanted with human IEQs (3000-4000) under the kidney capsule. This resulted in immediate establishment of normoglycemia (blood glucose<13.8 mM) [24], [32]. Islet allograft rejection, as evidenced by a rise in blood glucose above 20 mM, was observed in all hu-NSG mice by day 17 post-transplantation while normoglycemia remained stable over an observation of 90 days in NSG mice (6 animals for each group) (**Fig. 2A**). In order to demonstrate the functionality of transplanted islets *in vivo*, six mice with successful islet transplantations were selected to undergo unilateral left nephrectomy, which restored a hyperglycemic state within 24 hours (**Figure S3**). Human insulin levels in the sera from islet transplanted-hu-NSG mice correlated negatively with the levels of blood glucose (**Fig. 2B**).

**Figure 3:**
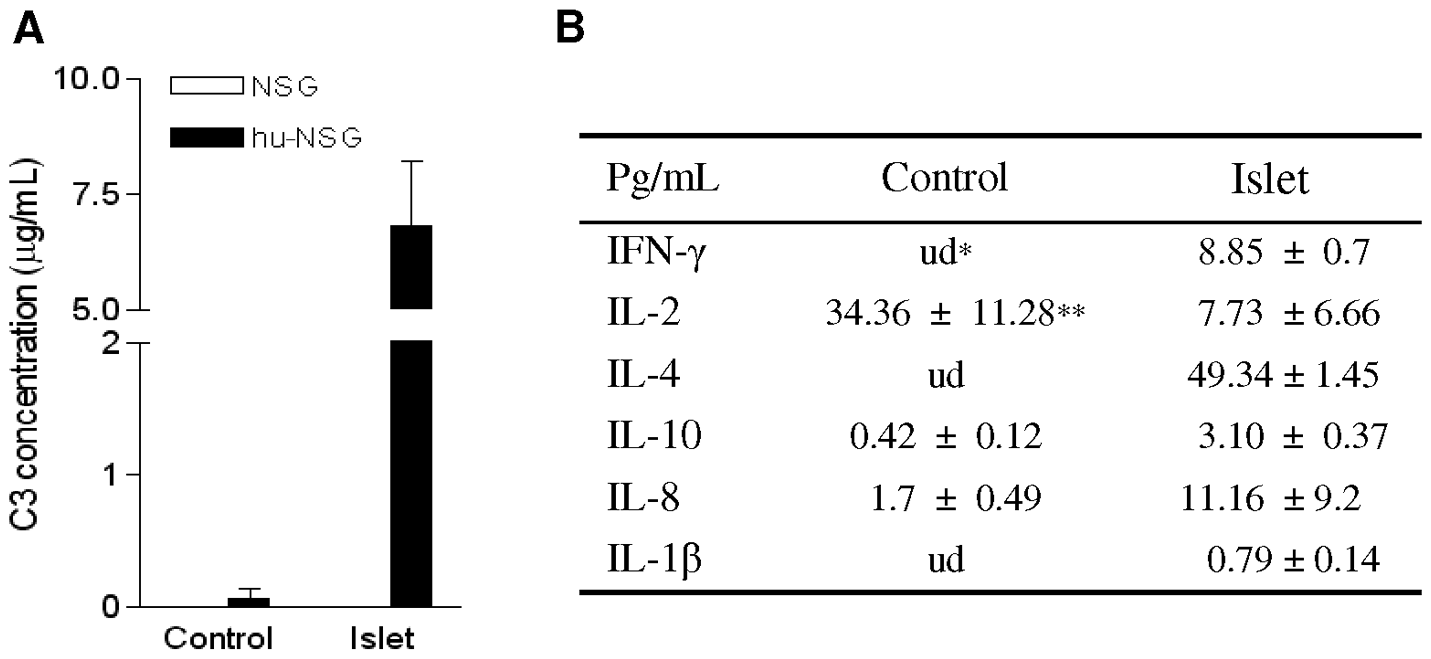
Human innate and adaptive immune responses mediate islet rejection in the hu-NSG mice. (A) Sera were analyzed by ELISA for human C3 levels in hu-NSG mice with/without human islet transplants or NSG mice with/without transplants (n = 3). Control: no islet transplant. (B) Sera were collected at the time of graft rejection. Cytokines were measured by cytokine bead array (n = 3). Control: sera from hu-NSG mice without islet transplant. *: undetectable. **: mean ± SD.

**Figure 4:**
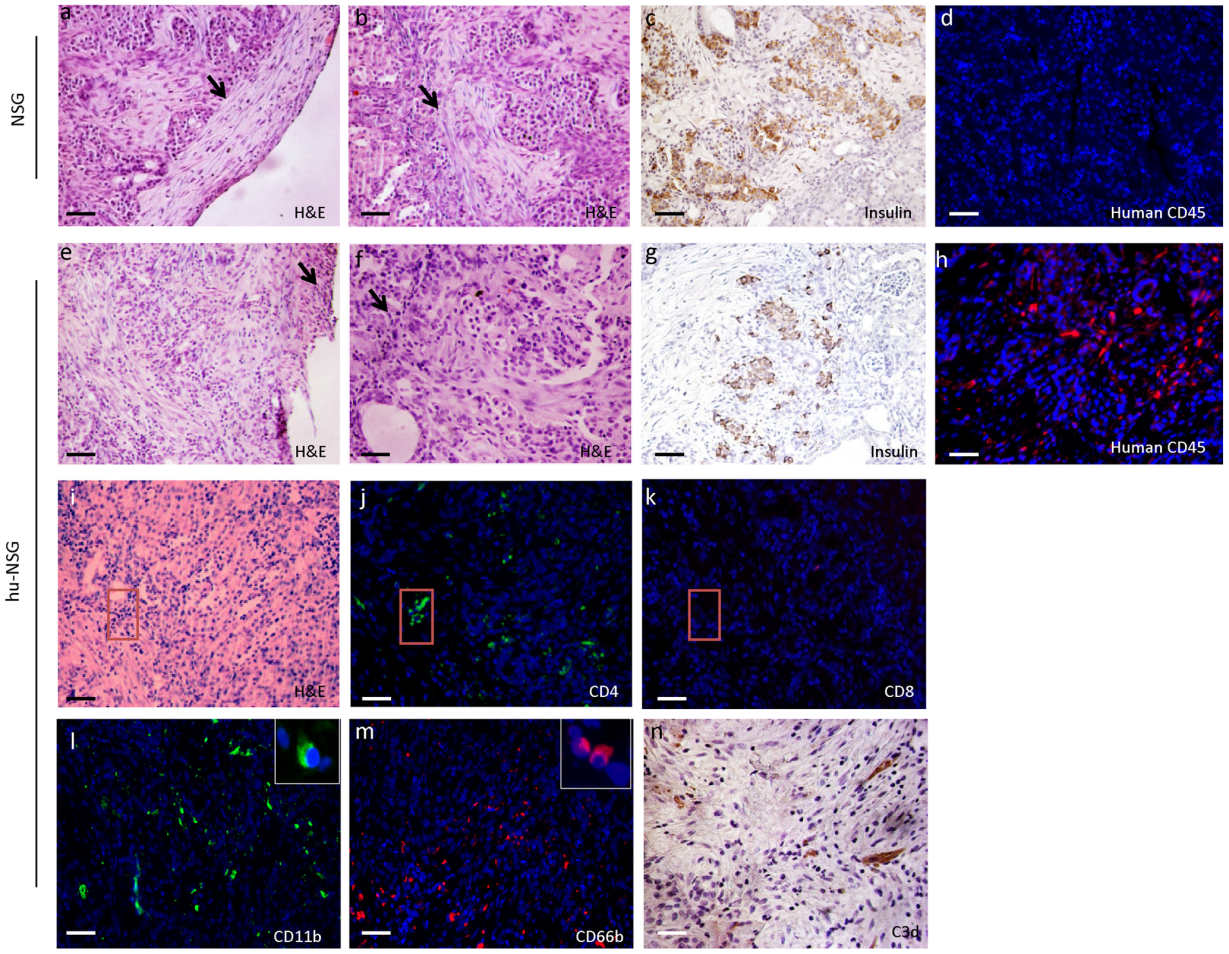
Infiltration of immune cells and C3 deposit in the grafted human islets. Tissue samples of islet allograft bearing kidney in hu-NSG mice were collected at the time of islet allograft rejection. Sections were stained with H & E (**a–b, e–f and i**) and antibodies to human antigens: insulin (**c** and **g**, brown), CD45 (**d and h**, green), CD4 (**j**, green), CD8 (**k**, red), CD11b (**l**, green), CD66b (**m**, red), and C3d (**n**, brown). Arrow indicates the kidney capsule or the area around islet grafts. Red square indicates the site of the infiltration. Scale bar: 100 µm.

Immune responses stimulated by islet allografts were analyzed in the sera and by histology at the time of rejection. Significant amounts of human C3 were detected in the sera of hu-NSG mice with islet transplants, but not in hu-NSG mice lacking islet transplants or in NSG mice with or without islet transplants (**Fig. 3A**). Human cytokines, including IFN-γ, IL-4 and IL-1β, were detected at pg/mL levels in the sera from the islet transplanted-hu-NSG mice but not in the hu-NSG without islet transplants (**Fig. 3B**). Histological analysis revealed damaged islet-structures with fewer insulin positive cells in mice with rejecting allografts (**Fig. 4c and g**). A large number of graft-infiltrating mononuclear cells (**Fig. 4e, f and i**) and human CD45^+^ cells (**Fig. 4h**) were detected in hu-NSG recipients, but not in NSG mice (**Fig. 4a, b and d**). A significant number of macrophages (CD11b^+^), neutrophils (CD66b^+^) and CD4^+^ T cells were recruited into the graft-bearing kidney tissue in hu-NSG mice (**Fig. 4l, m and j**). Very few, if any, CD8^+^ T cells were identified in hu-NSG mice (**Fig. 4k**). C3d deposition in the islet grafts was also observed (**Fig. 4n**). Altogether these results indicate that hu-NSG mice mount both innate and adaptive immune responses against transplanted human islet allografts.

### *Ex vivo* polyclonally expanded human Tregs delay human islet allograft rejection

Next, we investigated the impact of T_regs_ on the modulation of human islet allograft rejection. Human T_regs_ (CD4^+^CD25^+^) were isolated and expanded *in vitro* in the presence of rapamycin and IL-2 [14]. The T_reg_ lines were characterized phenotypically and functionally, prior to adoptive transfer into recipient mice. Flow cytometric analysis revealed that greater than 98% of CD4-gated T_regs_ expressed high levels of CD25 and FoxP3 (**Figure S4** (A)). T_regs_ displayed a marked suppressive ability as measured by CFSE dilution (**Figure S4** (B) and (C)).

**Figure 5:**
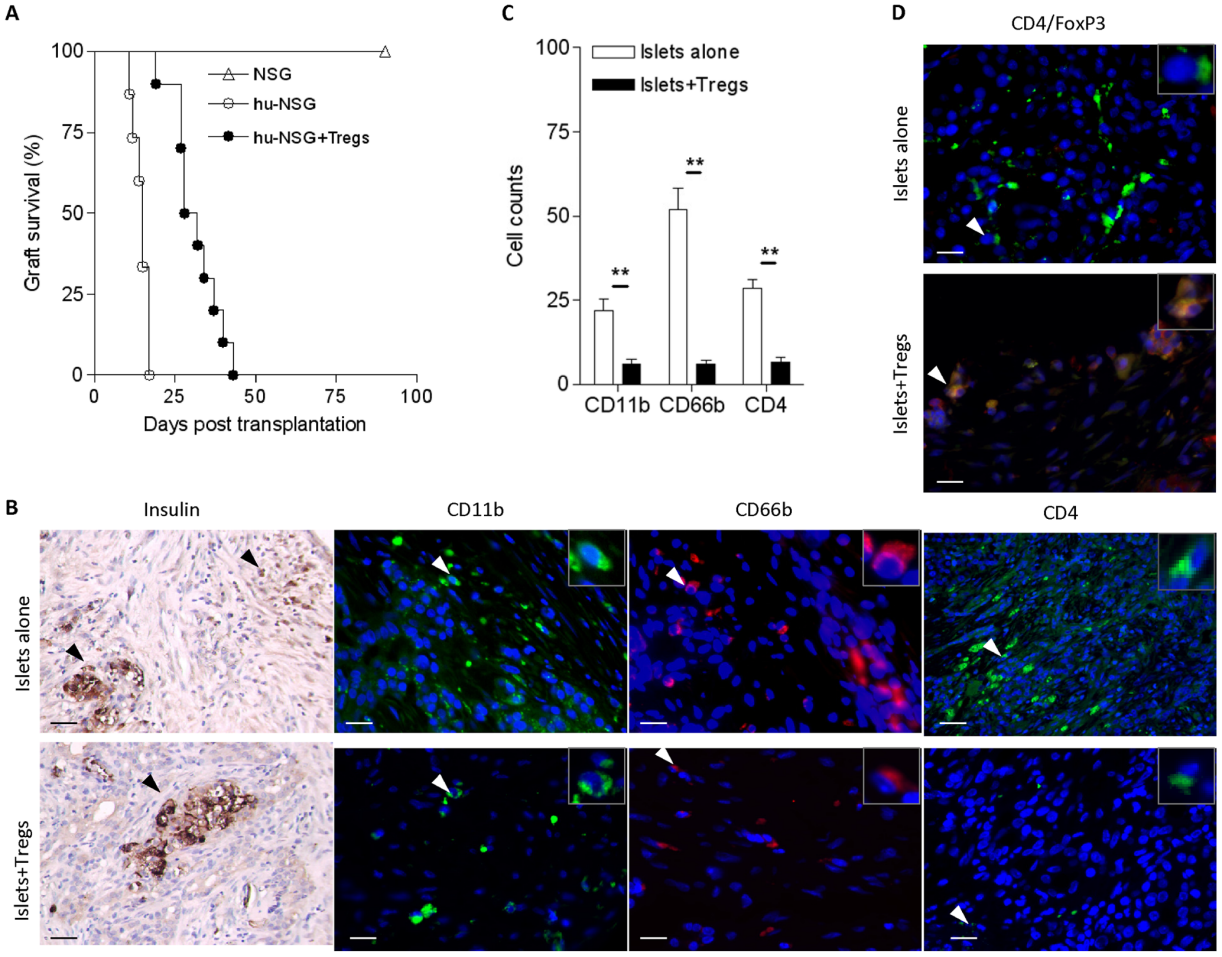
Adoptive transfer of *ex vivo* expanded T_regs_ protects human islet allograft from rejection. (A) Percentage graft survival in hu-NSG mice with or without T_reg_-treatments (n = 15 for islets alone group; n = 10 for T_regs_-treated group; Log-rank test, p = 0.0004). (B) Histological examination of islet grafts determined by immunostaining with antibodies for human antigens: insulin (brown), CD11b (green), CD66b (red), and CD4 (green). Nuclei were stained with DAPI (blue). Tissues were harvested at the time of islet allograft rejection in T_regs_-untreated animals and at day 21 post-islet transfer in T_reg_-treated animals. (C) Quantitative data analysis of islet graft immunostaining. Data represent results from three individual mice per group. (D) Identification of T_regs_ in the islet grafts. The harvested grafts were double-stained with FITC-conjugated CD4 (green) and TRITC-conjugated FoxP3 (red). CD4/FoxP3 double-positive cells are shown by yellow colour. Inset images show enlarged area indicated by a white arrow. Black arrow indicates islets grafts. **: p<0.01. Scale bar: 50 µm.

**Figure 6:**
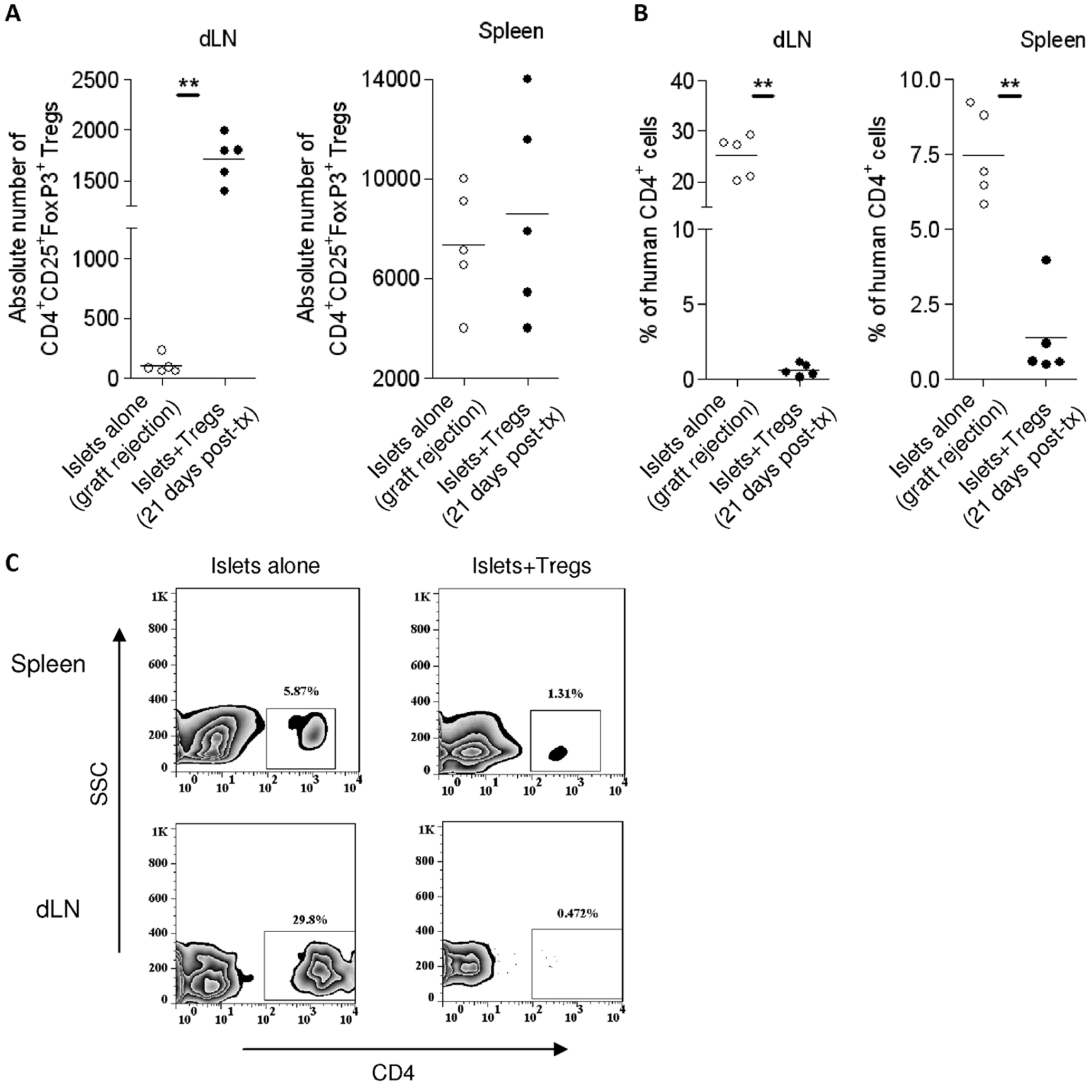
T_regs_ accumulate in the draining lymph nodes and inhibit CD4^+^ T cells in the draining lymph nodes and spleen. (A) The absolute number of CD4^+^CD25^+^FoxP3^+^ cells in the draining lymph nodes and spleens, at the time of graft rejection (islets alone group) or at day 21 post-islet transfer (islets+Tregs group) was determined by flow cytometry. (B) Percentage of cells positive for human CD4 in draining lymph nodes and spleens from (A). (C) Data are representative dot plots from three independent experiments. Plotted lines represent the mean (n = 5). **: p<0.01. dLN: draining lymph nodes. Post-tx: post islet transfer.

T_reg_ lines (6×10^6^) were then injected intravenously into hu-NSG mice at the time of islet transplant. Islet graft survival was monitored by measuring blood glucose levels in the sera of the mice. The results demonstrated that the transfer of *ex vivo* expanded T_regs_ prolonged the survival of islets (median survival time T_regs_-treated mice 32 days versus 17 days in mice receiving islets alone) (n = 15 for islets alone group; n = 10 for islets+T_regs_ group; **Fig. 5A**). To confirm that the prolonged graft survival was the result of T_reg_-mediated suppression, islet grafts were harvested at the time of graft-rejection for T_reg_-untreated recipients and at day 21 post-transplantation (animals with grafts undergoing rejection were excluded) for T_reg_-treated recipients. Histological analysis demonstrated that surviving grafts contained densely packed insulin-producing cells and intact islet structures, whereas grafts undergoing rejection exhibited destroyed lobular structure of islets with few remaining insulin positive-staining cells (**Fig. 5B**). The numbers of macrophages (CD11b^+^), neutrophils (CD66b^+^) and CD4^+^ T cells infiltrating the grafts were markedly reduced in T_reg_-treated animals, compared with T_reg_-untreated recipients (n = 3 for each group) (**Fig. 5B and C**). These results suggest a potent suppression of innate and CD4^+^ T cell-mediated immune responses by T_regs_. The improved histology correlated with the presence of T_regs_ (CD4^+^FoxP3^+^) in the graft of T_reg_-treated animals (**Fig. 5D**) and higher numbers of T_regs_ in the draining lymph nodes (**Fig. 6A**). Lower numbers of total CD4^+^ T cells were observed in the spleens and draining lymph nodes of these mice compared to T_reg_-untreated mice (**Fig. 6B and C**), whereas no significant differences were found in the number of CD4^+^CD25^+^FoxP3^+^ cells in the spleens between the two groups of mice (**Fig. 6A**).

### *Ex vivo* expanded human Tregs down regulate MCP-1 expression by islets

**Figure 7:**
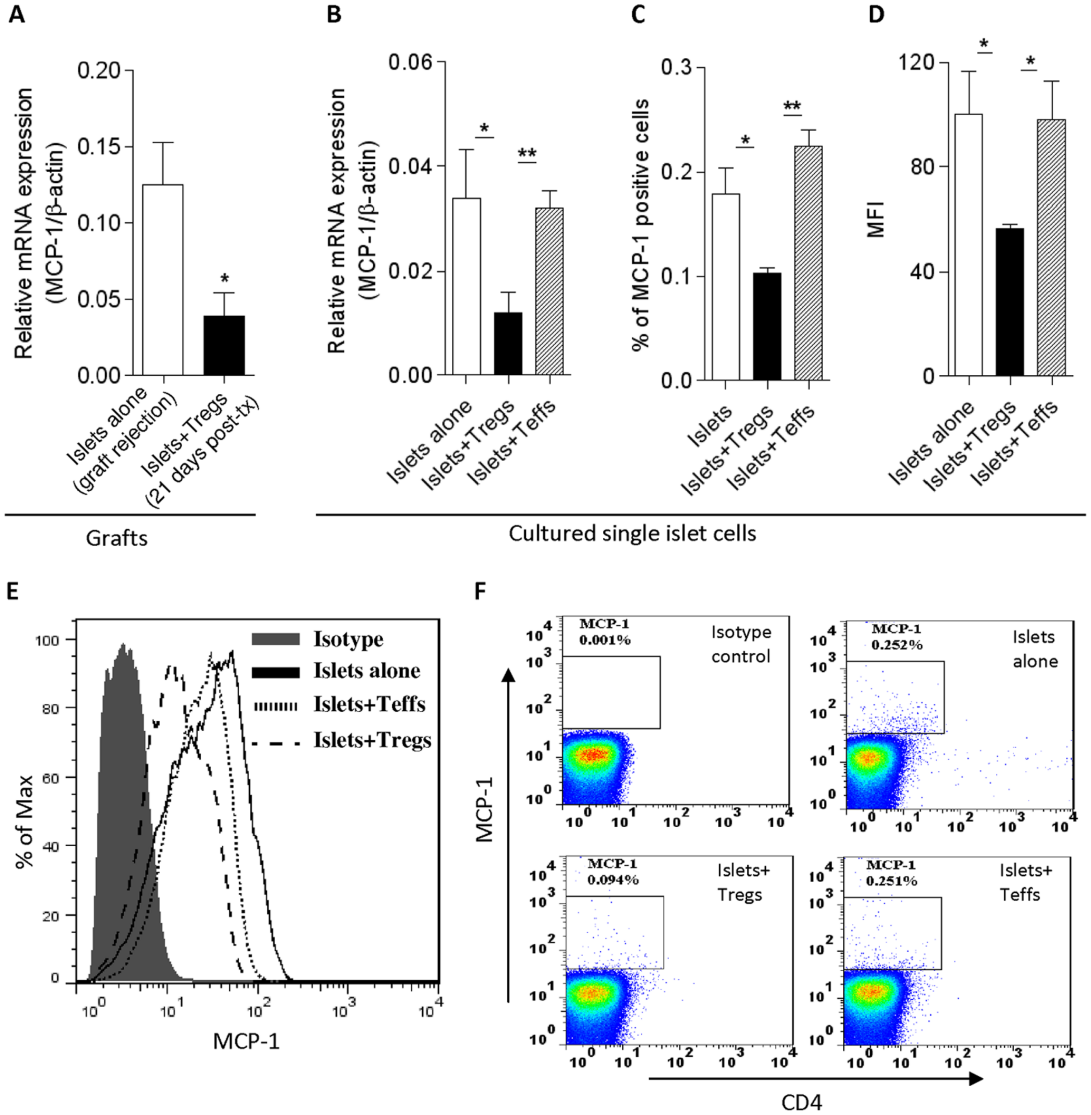
T_regs_ reduced MCP-1 production *in vitro* and *in vivo*. (A) MCP-1 expression in islet grafts at the time of rejection (islets alone group) or at day 21 post-islet transfer (islets+T_regs_ group) was measured by real-time RT-PCR (n = 5). β-actin was used as the endogenous control. (B) The expression of mRNA was analyzed by real-time RT-PCR in islet cells co-cultured with T_regs_ for three days. (C–D) Cumulative data (mean ± SD) for MCP-1 expression in cultured islet cells. MFI: mean fluorescence intensity. (E–F) Representative plots from 4 independent experiments are shown. *: p<0.05; **: p<0.01. Post-tx: post islet transfer.

**Figure 8:**
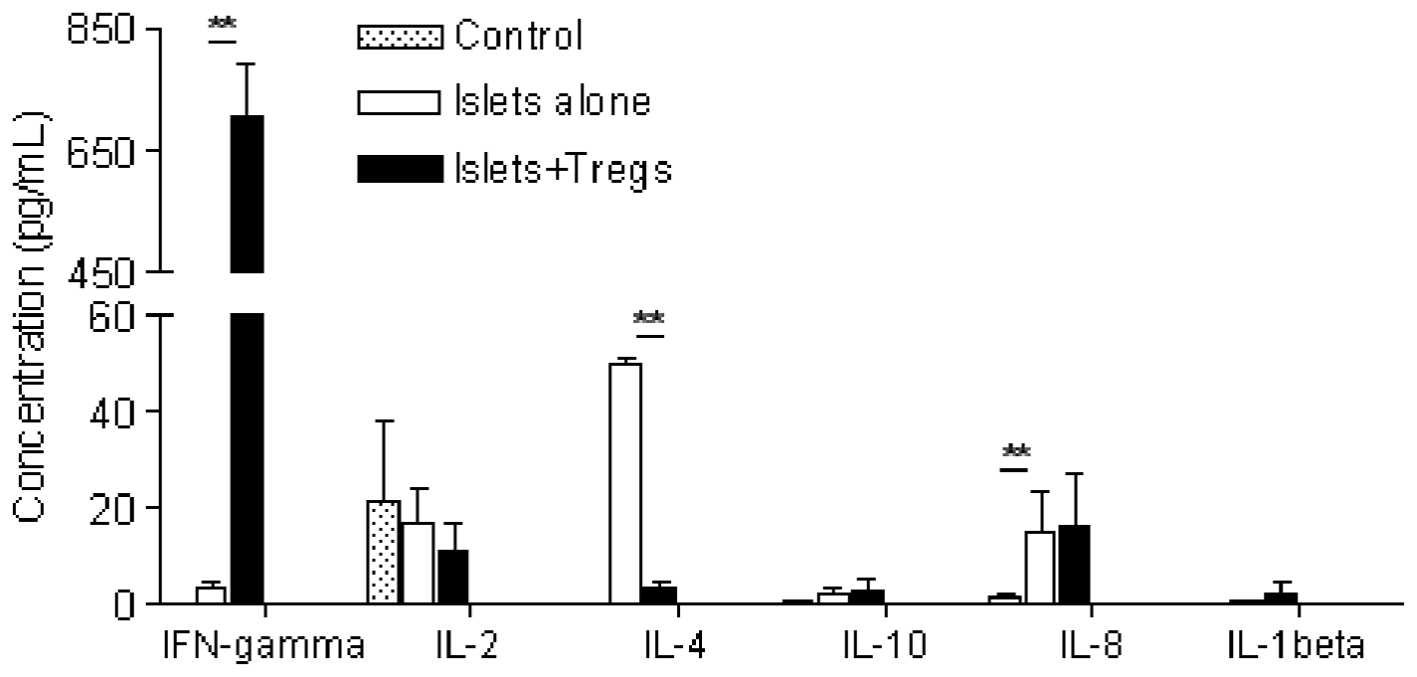
T_regs_ regulate human cytokine production in islet-transplanted hu-NSG mice. Sera were collected at the time of rejection (islets alone group) or at day 21 post-islet transfer (islets+T_regs_ group). Cytokines were measured by cytokine bead array (n = 3). Control: sera from hu-NSG mice without islet transplant. **: p<0.01.

Having observed a decreased infiltration of macrophages (CD11b^+^) and neutrophils (CD66b^+^) in the islet grafts of hu-NSG mice and an increase in T_regs_ in the draining lymph nodes following adoptive transfer of T_regs_, we chose to focus on the mechanisms behind the regulation of innate cell infiltration. Specifically, we investigated the possibility that T_regs_ exhibited suppression of innate cells by regulating the expression of MCP-1, which is secreted by islet cells and is responsible for macrophage/neutrophil recruitment [33], [34]. Islet allografts were harvested at the time of rejection in the T_reg_-untreated group and at day 21 post-transplantation in the T_reg_-treated group. RT-PCR analysis showed that the level of MCP-1 gene transcription in T_reg_-treated animals was reduced by 71% compared with T_reg_-untreated animals (**Fig. 7A**). To test the direct effect of T_regs_ on MCP-1 production by islets, T_regs_ were co-cultured with freshly isolated single islet cells *in vitro*. Consistent with the *in vivo* findings, T_regs_ reduced MCP-1 transcription in the cultured islet cells (**Fig. 7B**). In contrast, autologous T_effs_ co-cultured with islet cells had no effect on MCP-1 expression (**Fig. 7B**). Flow cytometric analysis confirmed that T_regs_ significantly decreased the number of MCP-1 positive cells in islet cells (**Fig. 7C and F**) and the expression of MCP-1 by islet cells during co-culture with T_regs_ (**Fig. 7D and E**). Interestingly, when the cytokine profile was analyzed in the sera of T_reg-_treated animals, IFN-γ was remarkably increased while IL-4 was significantly decreased (**Fig. 8**). Compared with controls, IL-8 was significantly elevated in serum of islet-transplanted hu-NSG mice (**Fig. 8**).

## Discussion

The data presented here show that hu-NSG mice develop a functional human immune system [26], [29], and for the first time we demonstrate the utility of hu-NSG mice for studying innate immune responses to human islet allografts. Our study provides evidence that *ex vivo* expanded human T_regs_ delay human islet allograft rejection in hu-NSG mice, via a mechanism involving reduced expression of MCP-1 by engrafted islets and inhibition of infiltration by macrophages, neutrophils and CD4^+^ T cells.

Although immunodeficient mice allow engraftment of human hematopoietic stem cells, it has not yet been possible to achieve completely normal human hematopoiesis in any of the currently available immunodeficient mouse strains [28], [29]. The hu-NSG mice generated in this study were approximately 30% chimeric with human cells (CD45^+^ cells) including T- and B-cells and subsets of various immune cells that are present in the spleen: these include T_regs_ (CD4^+^CD25^+^FoxP3^+^), monocytes/macrophages (CD14^+^), DC (CD11c^+^) and NK cells (CD56^+^CD16^+^), indicating reconstitution of human lymphohematopoiesis in these mice. We noted human CD3 T cells engraftment in hu-NSG mice 12-16 weeks after CD34^+^ cell transfer, which was earlier than other reports [35]. This may be due to differences in experimental conditions and protocols used to engraft human stem cells, including the choice of recipient age and source of human CD34^+^ cells [35].

The engrafted human T cells in our system were able to produce cytokines including IL-2, TNF-α, TNF-β, IL-8 and IL-1β and to respond to polyclonal CD3/CD28 stimulation, although less efficiently than in adult blood, suggesting partial function of engrafted T cells [26], [29]–[31], [36], [37]. Confirming published work [26], [30], [31], [36]–[38], an impaired B cell response was also observed in these animals, showing inefficient production of antigen-specific IgG. This is believed to be due to defective interaction between human B and T cells [26], [30], [31], [36]–[38], resulting from a lack of human leukocyte antigen (HLA) expression in the mouse thymus [26], [38]. This possibility has been supported by the improved antigen-specific human T cell and antibody responses achieved when human CD34^+^ cells were injected in immunodeficient mice that expressed HLA molecules either by transgenesis [38], [39] or following transplantation of human thymus [40]. Nevertheless, although NSG mice receiving CD34^+^ cells failed to develop efficient antigen-specific immune responses [26], [30], [31], [36], [37], we found that, following immunization, engrafted human T cells responded to alloantigens, as shown by ^3^H-thymidine incorporation in an *in vitro* culture of CD4^+^ cells from the spleen. These findings do not exclude the possibility that other antigen recognition pathways exist [28], thus there remains a need to assess the major histocompatiblity complex (MHC) restriction of human T cells in this animal model.

The importance of the innate immune system is currently poorly understood in this model of human allograft rejection. The presence of C3d deposition and infiltration of macrophages (CD11b^+^) and neutrophils (CD66b^+^) into rejecting islet allografts in these animals supports the idea that our system might be useful for the study of innate immune responses to allografts. This view is further strengthened by the detection of human C3 in the sera of the islet-transplanted hu-NSG mice generated in this study, which we speculate was locally produced by infiltrating human inflammatory cells [41], [42]. Indeed, local immune cell-derived production of complement emerges as a key mediator of complement's impact on adaptive immune responses [42].

One of the advantages of CD34^+^ cells-reconstituted humanized mice is that they lack graft-versus-host disease (GvHD) [43]. By rendering mice diabetic before islet transplantation, we have been able to monitor the islet allograft rejection spontaneously mediated by hu-NSG mice, evidenced by raising blood glucose. This contrasts with PBMC-reconstituted NSG (huPBL-NSG) mice, which died from GvHD within the first 30 days and provided only 4-5 week window of opportunity of the human immune responses [32]. As a consequence, in the huPBL-NSG mice, rejection is often determined *post-facto* by histopathology, making the huPBL-NSG mouse model difficult to use for interventional studies [32]. In keeping with blood glucose data, histological examination showed a significant human CD45^+^ leukocytes infiltrate and islet destruction in islet allograft, suggesting human immune-mediated rejection in our system. These data are inconsistent with the experiments using human CD34^+^ cells-reconstituted Balb/cRag2^−/−^γc^−/−^ mice [44]. The explanation for this discrepancy might lie in the difference in the mouse strains [45], [46]. Indeed NSG mice, with NOD (nobese diabetic) background, allow better hematopoietic engraftment than do Balb/cRag2^−/−^γc^−/−^ mice because the NOD variant of signal regulatory protein-α (SIRP-α) binds human CD47, whereas Balb/cRag2^−/−^γc^−/−^ mouse SIRP-α does not [18]. Binding of SIRP-α expressed on mouse macrophages to human CD47 is critical for the development of human hematopoiesis *in vivo* and *Sirpa* polymorphism has been identified as a new genetic determinant of human hematopoietic stem cell engraftment [18].

We have here tested for the first time the impact of *ex vivo* expanded human T_regs_ on the innate immune responses to human islet allograft in the humanized mice. Several groups have developed an optimal protocol for expanding T_regs_ for therapeutic use [47]–[49]. T_regs_ used in current study were expanded in the presence of CD3/CD28 beads and rapamycin as we previously published [14]. Rapamycin prevents outgrowth of contaminating non-regulatory cells, enhances T_reg_ survival and expands the most stable subpopulation of T_regs_ [49]. In the current study, we observed that in T_regs_-treated group, islet rejection was delayed for 15 days. Histological analysis demonstrated that there was significantly less infiltrating macrophages, neutrophils and CD4^+^ T cells with preservation of islet structure in the grafts from T_reg_-treated animals, suggesting that suppressive properties of T_regs_ are not limited to effects on T-cell responses but also include inhibition of pathology mediated by cells of the innate immune system system [50], [51]. Our demonstration is in agreement with recent findings suggesting that expanded human T_regs_ can prevent rejection of porcine islet xenograft in huPBL-NSG mice [52] and human islet allograft in PBMC-reconstituted Balb/cRag2^−/−^γc^−/−^ mice [24]. Similar results were also observed in the study of capacity of T_regs_ to interfere with the innate immune responses from other groups, including ours [53], in murine models of infectious diseases [54], skin transplantation [53] and islet engraftment [50]. Whether the inhibition of CD4^+^ T cell recruitment to the islet allograft was due to a direct effect of T_regs_ on T cells or the consequence of the action of T_regs_ on the innate immune system, as recently suggested [50], needs further investigation.

Our data also showed that T_regs_ detected by double staining of CD4 and FoxP3 were not present in a large numbers in the graft at day 21 post-transfer in T_regs_-treated group, suggesting that they may have had an initial effect on the islet, and then migrated to draining lymph nodes [50] [24]. This speculation was confirmed by our findings that the absolute numbers of T_regs_ was significantly higher in draining lymph nodes, along with significant decrease of CD4^+^ T cells in spleen and draining lymph nodes from T_reg_-treated animals when compared with in T_regs_-untreated animals. In line with our findings, T_regs_ have been demonstrated to sequentially migrate from the site of tissue inflammation to the draining lymph nodes to suppress alloimmune responses in a mouse islet allograft model [55].

The ability of T_regs_ to inhibit a range of immune responses and target a variety of cells suggests that they use multiple different effector mechanisms [56]. In our study, T_regs_ directly interacted with pancreatic islets, in the absence of effect T-cells, and inhibited the innate responses of islets for chemokine MCP-1 production *in vitro*. Consistent with our *in vitro* data, adaptive transfer of T_regs_ resulted in decreased expression of MCP-1 on islet graft *in vivo*. This finding may partly explain the mechanism underlying inhibiting of infiltration by T_regs_ in the graft. Furthermore, elevated serum levels of IFN-γ in the T_regs_-treated animals suggest the contribution of IFN-γ to regulation of T_regs_ as previously published [57], although additional work needs to be done to prove that IFN-γ is essential to the delay of islet rejection [57]. IFN-γ is secreted by Th1 cells and conventionally thought to be responsible for driving cell-mediated immune responses. However, in recent years the promoting effects of IFN-γ on the development of Th1-controlling T_regs_ has drawn attention from various groups [58], [59]. The early production of IFN-γ by induced T_regs_ following reencounter with antigen has been demonstrated to prevent the initiation of aggressive immune responses, including graft rejection, by controlling T-cell effector mechanisms [60]. In contrast to our findings, one report showed decreased IFN-γ levels in the sera from T_regs_-treated mice [52]. The difference in IFN-γ levels might be the different type of human hematopoietic cells engrafted through reconstitution with either CD34^+^ cells or PBMC [52]. In addition, IL-4, which can up-regulate MCP-1 expression in a variety of cells [61], was remarkably decreased in T_reg_-treated animals. These data suggest that the balance between these two cytokines might contribute to the regulation of cellular MCP-1 expression [62].

In conclusion, the present study provides evidence that *ex vivo* expanded human T_regs_ prolong islet allograft survival via inhibiting infiltration of macrophages, neutrophils and CD4^+^ T cells possibly through a MCP-1-dependent mechanism. These data may contribute to the development of clinical strategies for the generation of T_reg_ therapy to control human islet rejection. Also, we reveal for the first time that CD34^+^ stem cells-reconstituted NSG mouse model might offer a tool for pre-clinical evaluation of human innate immune responses to human allografts.

## Supporting Information

Figure S1 **Assessment of human T cell function in hu-NSG mice.** (A) Splenocytes from hu-NSG mice and PBMC from healthy donors (AdPBMC) were stimulated with CD3/CD28 beads for 3 days. T cell function was determined by ^3^H-thymidine uptake (n = 6). (B) Human CD4^+^ T cells isolated from hu-NSG mice immunized with 10^7^ PBMC (specific) were cultured with the same PBMC and with third party PBMC for 5 days. T cell function was assessed in MLRs (n = 3). The insert shows the spleens from a representative immunized (left) and non-immunized mouse (right).
(TIF)

Figure S2 **Production of human immunoglobins by human B cells in hu-NSG mice.** (A) Human IgM and IgG were determined by ELISA in sera from hu-NSG mice 12–16 weeks after CD34^+^ cell reconstitution and in supernatants of B cells cultured in the presence of IL-2 and IL-21. (B) Keyhole limpet haemocyanin (KLH)-specific human IgM or IgG were determined in the sera from hu-NSG mice immunized with KLH. Sera from non-immunized hu-NSG mice were used as control. (n = 3). For the B cell culture, a representative result of three independent cultures is shown. NSG: sera from NSG mice without CD34^+^ cell reconstitution; Nil: supernatant from the B cell culture in the absence of IL-2 and IL-21.
(TIF)

Figure S3 **Assessment of grafted human islet in hu-NSG mice.** Human islet equivalents (IEQs, 3000–4000) were transplanted into the kidney capsule of chemically-induced diabetic mice and blood glucose was measured. The islets were removed via unilateral nephrectomy when establishment of normoglycemia (blood glucose<13.8 mM) had been confirmed in hu-NSG mice and at day 30 post-islet transfer in NSG mice. The function of human islet grafts was evaluated by measuring blood glucose levels. All mice returned rapidly to a hyperglycemic state. Data shown are representative examples from 3 animals in each group. STZ: streptozotocin.
(TIF)

Figure S4 **The phenotype and functional analysis of** ***ex vivo*** **expanded human T_regs_.** (A) Representative flow cytometric analysis of the expression of CD25 and FoxP3 molecules by T_regs_ (cells were gated on human CD4 expression). Data are from one out of three independent experiments with T_regs_ derived from three individual donors. (B) CFSE–labelled CD4^+^CD25^−^ cells (responders) were co-cultured with serial dilutions of expanded autologous T_regs_ in the presence of anti-CD3/CD28 beads for 5 days. Flow cytometric analysis was performed to assess cellular proliferation by CFSE dilution. (C) Quantitative analysis of T_regs_ suppressive function. The data shown are representative of three independent experiments.
(TIF)

Table S1 **HLA typing.**
(TIF)
