# Correction: *Ex Vivo* Expanded Human Regulatory T Cells Delay Islet Allograft Rejection via Inhibiting Islet-Derived Monocyte Chemoattractant Protein-1 Production in CD34^+^ Stem Cells-Reconstituted NOD-*scid IL2rγ^null^* Mice

**DOI:** 10.1371/journal.pone.0098722

**Published:** 2014-05-23

**Authors:** The PLOS ONE Staff

There are errors in the Author Contributions section. The correct Author Contributions are: Conceived and designed the experiments: FX LM VM RL GL. Performed the experiments: FX LM. Analyzed the data: FX LM. Contributed reagents/materials/analysis tools: MZ GH. Wrote the paper: FX. Helped in designing the experiments: GH AD. Reviewed/edited the manuscript: LM AD RL GL.

## References

[1] Xiao F, Ma L, Zhao M, Huang G, Mirenda V, et al (2014) *Ex Vivo* Expanded Human Regulatory T Cells Delay Islet Allograft Rejection via Inhibiting Islet-Derived Monocyte Chemoattractant Protein-1 Production in CD34^+^ Stem Cells-Reconstituted NOD-*scid IL2rγ^null^* Mice. PLoS ONE 9(3): e90387 doi:10.1371/journal.pone.0090387 24594640 10.1371/journal.pone.0090387 PMC3940883

