# Supplementary figures and images for: Ex Vivo Expanded Human Regulatory T Cells Delay Islet Allograft Rejection via Inhibiting Islet-Derived Monocyte Chemoattractant Protein-1 Production in CD34+ Stem Cells-Reconstituted NOD-scid IL2rγnull Mice
Source: PLoS One. 2014 Mar 3;9(3):e90387. doi: 10.1371/journal.pone.0090387 (PMC3940883; doi:10.1371/journal.pone.0090387)

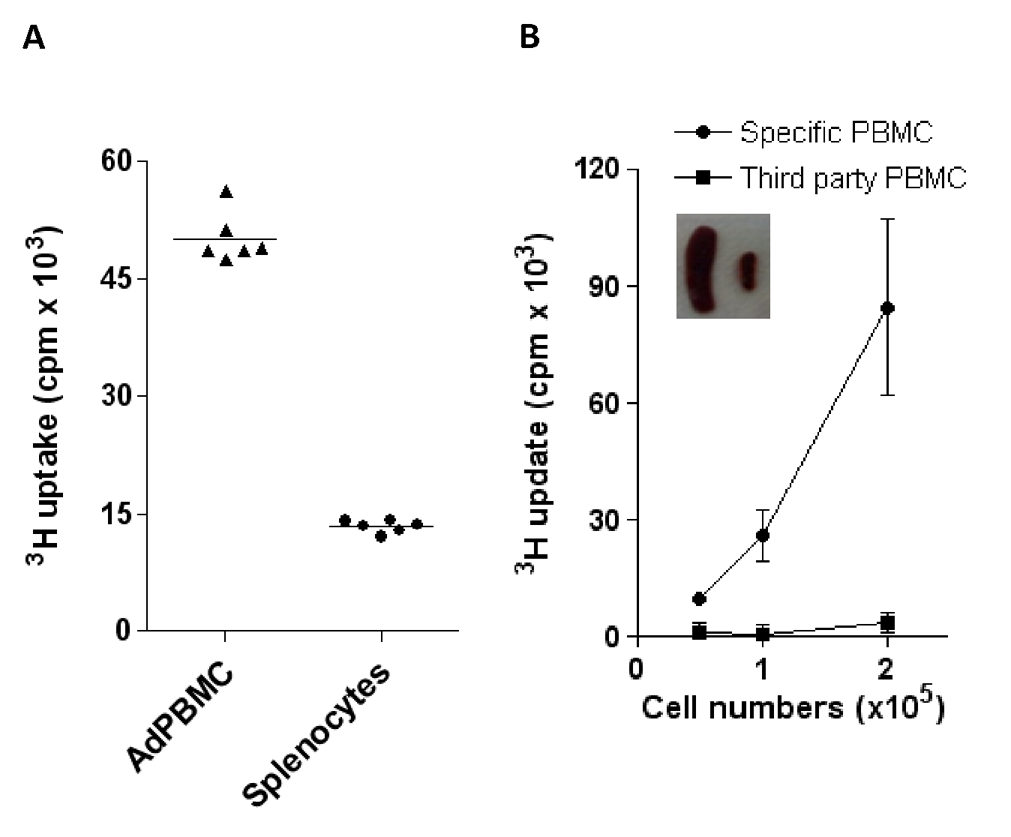

Supplement: Figure S1 — Assessment of human T cell function in hu-NSG mice. (A) Splenocytes from hu-NSG mice and PBMC from healthy donors (AdPBMC) were stimulated with CD3/CD28 beads for 3 days. T cell function was determined by 3H-thymidine uptake (n = 6). (B) Human CD4+ T cells isolated from hu-NSG mice immunized with 107 PBMC (specific) were cultured with the same PBMC and with third party PBMC for 5 days. T cell function was assessed in MLRs (n = 3). The insert shows the spleens from a representative immunized (left) and non-immunized mouse (right). (TIF) [file pone.0090387.s001.tif]

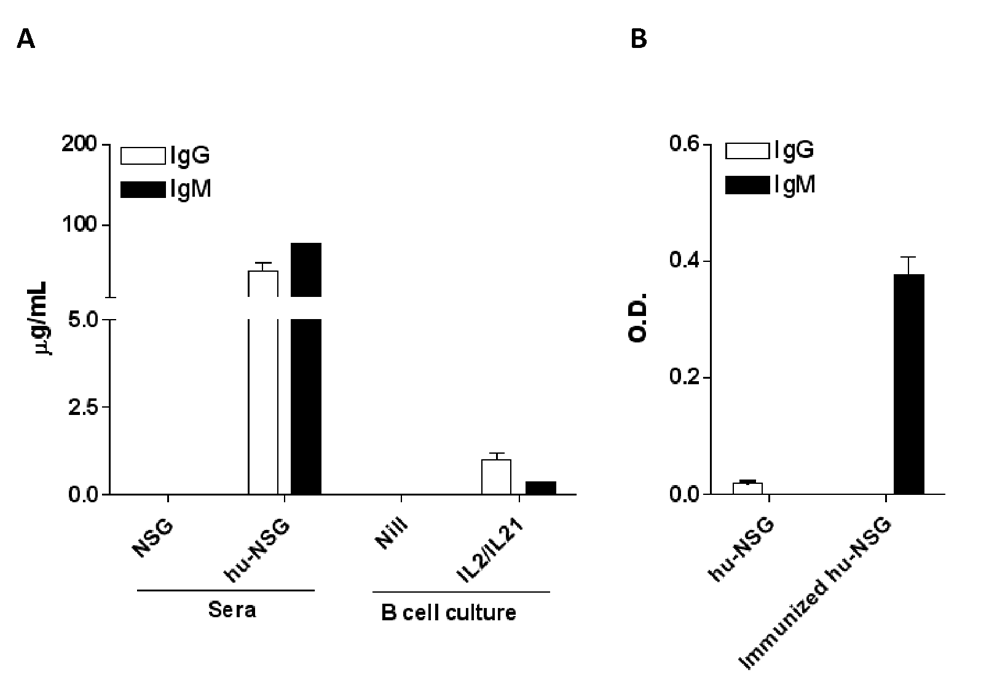

Supplement: Figure S2 — Production of human immunoglobins by human B cells in hu-NSG mice. (A) Human IgM and IgG were determined by ELISA in sera from hu-NSG mice 12–16 weeks after CD34+ cell reconstitution and in supernatants of B cells cultured in the presence of IL-2 and IL-21. (B) Keyhole limpet haemocyanin (KLH)-specific human IgM or IgG were determined in the sera from hu-NSG mice immunized with KLH. Sera from non-immunized hu-NSG mice were used as control. (n = 3). For the B cell culture, a representative result of three independent cultures is shown. NSG: sera from NSG mice without CD34+ cell reconstitution; Nil: supernatant from the B cell culture in the absence of IL-2 and IL-21. (TIF) [file pone.0090387.s002.tif]

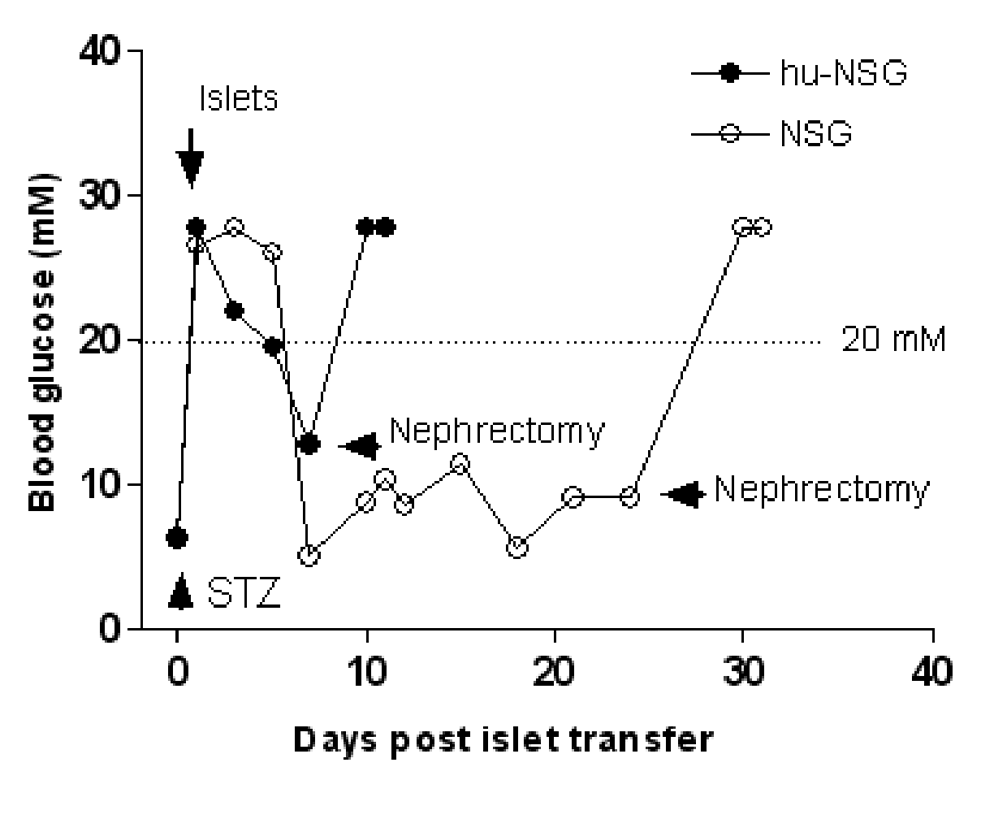

Supplement: Figure S3 — Assessment of grafted human islet in hu-NSG mice. Human islet equivalents (IEQs, 3000–4000) were transplanted into the kidney capsule of chemically-induced diabetic mice and blood glucose was measured. The islets were removed via unilateral nephrectomy when establishment of normoglycemia (blood glucose<13.8 mM) had been confirmed in hu-NSG mice and at day 30 post-islet transfer in NSG mice. The function of human islet grafts was evaluated by measuring blood glucose levels. All mice returned rapidly to a hyperglycemic state. Data shown are representative examples from 3 animals in each group. STZ: streptozotocin. (TIF) [file pone.0090387.s003.tif]

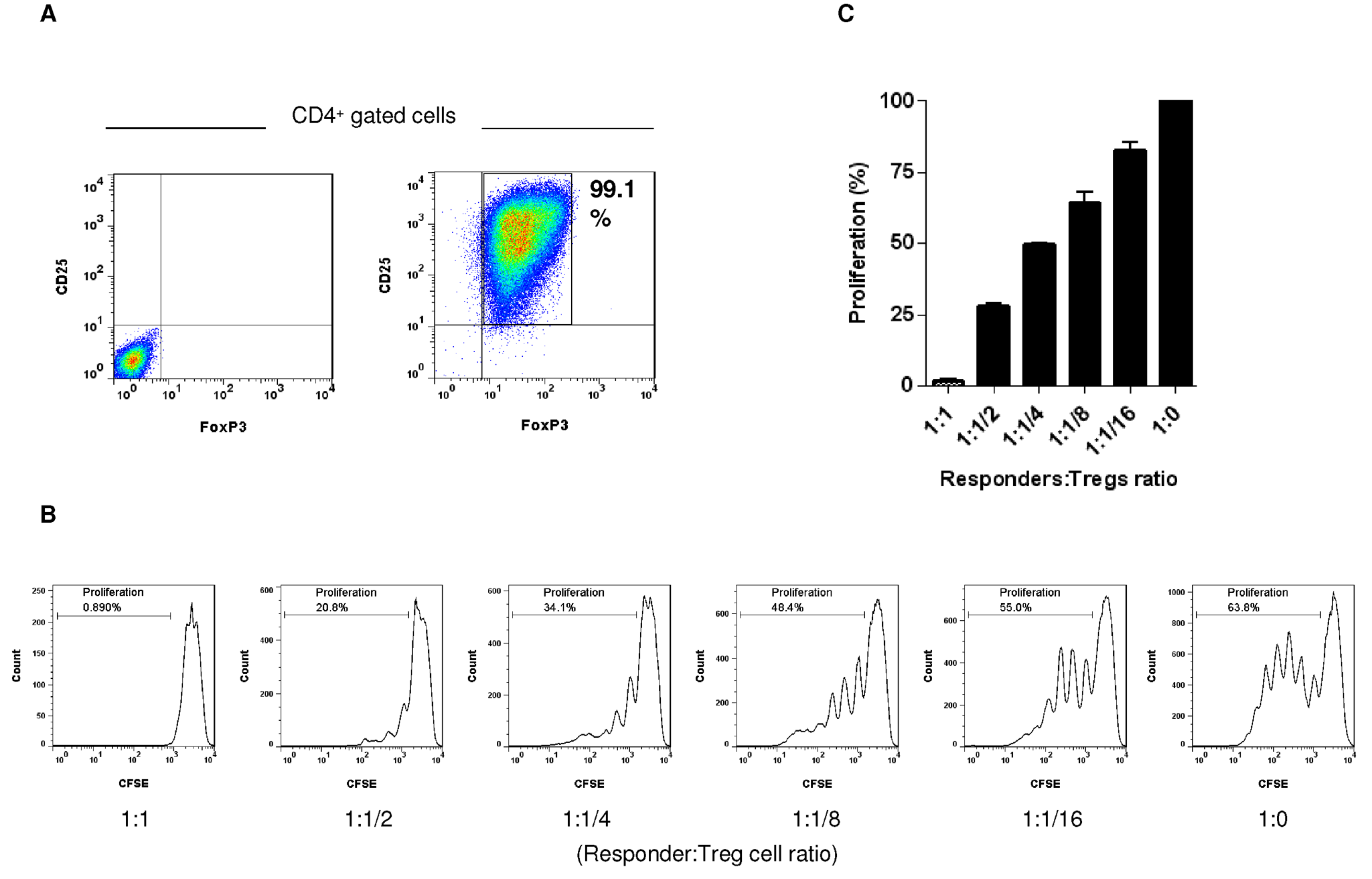

Supplement: Figure S4 — The phenotype and functional analysis of ex vivo expanded human Tregs. (A) Representative flow cytometric analysis of the expression of CD25 and FoxP3 molecules by Tregs (cells were gated on human CD4 expression). Data are from one out of three independent experiments with Tregs derived from three individual donors. (B) CFSE–labelled CD4+CD25− cells (responders) were co-cultured with serial dilutions of expanded autologous Tregs in the presence of anti-CD3/CD28 beads for 5 days. Flow cytometric analysis was performed to assess cellular proliferation by CFSE dilution. (C) Quantitative analysis of Tregs suppressive function. The data shown are representative of three independent experiments. (TIF) [file pone.0090387.s004.tif]

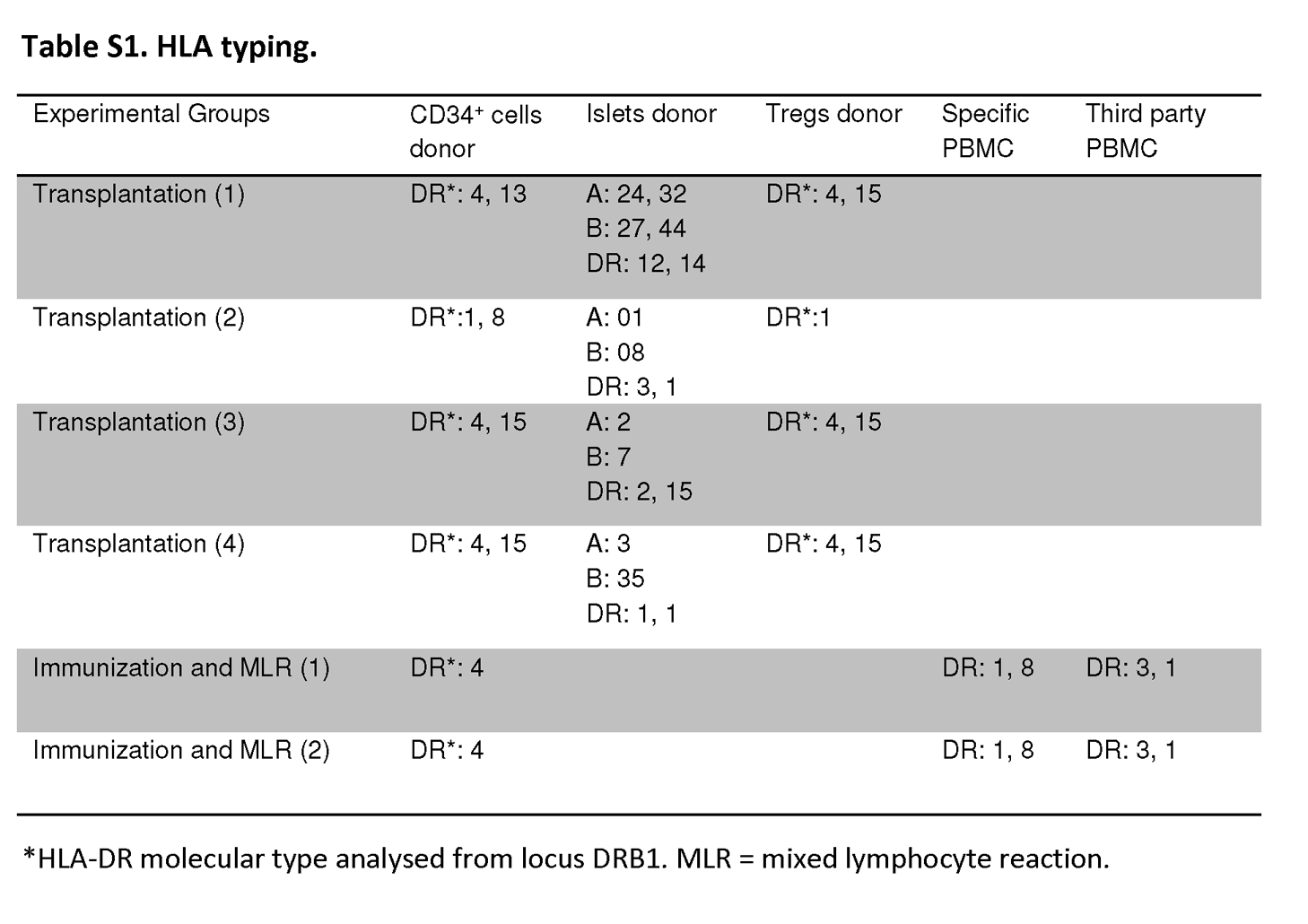

Supplement: Table S1 — HLA typing. (TIF) [file pone.0090387.s005.tif]
